# Formaldehyde in Root-Canals

**Published:** 1903-10-15

**Authors:** B. F. Thielen


### FORMALDEHYDE IN ROOT-CANALS.

For more than a year I have used this preparation in the treatment of root-canals, and success has attended its use in every case. In all cases of immediate extirpation of the pulp, after the canals have been thoroughly cleansed, an application of formaldehyde, forty percent solution, is made to the canals after which they are dried and immediately filled.

Where a pulp has died and become putrescent, it is removed, the canals thoroughly cleansed with an application of ammonia water followed by warm water, then, after thoroughly drying, the formaldehyde is applied, after which the canals are sealed for twenty-four or forty-eighthours.

Then, in the majority of cases, the putrescent odor has disappeared and the canals are ready for permanent filling. To give an example, only a few days since, a patient came in, complaining of having spent a restless night, due to an aching tooth. An examination revealed the fact that an upper second bicuspid was in a state of incipient abscess. An opening was made into the pulp chamber, followed by a slight flow of pus. This was evacuated, the canals thoroughly cleansed, and formaldehyde applied. It was then temporarily sealed and a counter-irritant was applied to the gum. This was about 10 a. m. The next day at the same hour the patient returned, had had no further pain, soreness to percussion was gone; the tooth was opened up, the characteristic odor had disappeared, the canals were filled, and the tooth crowned.

Some who have written on this subject recommend that the drug be used in milder solution. I have always used the forty percent solution, and in no case have there appeared any untoward effect.—B. F. Thielen, in *Texas Journal.*

